# Effects of the LEARNS model-based self-management education on short-term treatment outcomes among newly diagnosed tuberculosis patients: a randomized controlled trial

**DOI:** 10.3389/fpubh.2026.1861683

**Published:** 2026-08-11

**Authors:** Huijuan Wang, Rong Yong, Meng Hao, Xiaohui Liu

**Affiliations:** 1 The Fourth People's Hospital of Ningxia Hui Autonomous Region, Yinchuan, China; 2 School of Nursing, Ningxia Medical University, Yinchuan, China

**Keywords:** LEARNS model, newly diagnosed, randomized controlled trial, self-management, tuberculosis

## Abstract

**Background and Objectives:**

Newly diagnosed patients with pulmonary tuberculosis usually have insufficient self-management ability. LEARNS model-based health education has been shown to significantly improve patients' self-management and treatment outcomes. This randomized controlled trial aimed to evaluate the effects of the LEARNS model-based self-management intervention on six indicators among patients with newly diagnosed tuberculosis: self-management, tuberculosis prevention and control knowledge, anxiety, depression, social support, and treatment efficacy.

**Methods:**

This prospective, two-arm, parallel-group randomized controlled trial was registered (ChiCTR2400090561). Seventy-two inpatients with newly diagnosed pulmonary tuberculosis were enrolled from the tuberculosis department of a tertiary hospital in Ningxia between May and July 2024. Participants were randomly assigned to receive either routine tuberculosis self-management education (control group) or standardized LEARNS model-based self-management education (intervention group). Self-management was defined as the primary outcome. Secondary outcomes encompassed tuberculosis prevention and control knowledge, perceived social support, anxiety, depression, sputum smear and culture conversion rates, and chest CT lesion improvement rates. All participants underwent a 2-month integrated intervention program involving inpatient-based education and continuous post-discharge follow-up. All study outcomes were evaluated 3 months after program completion. All intention-to-treat analyses were performed using generalized estimating equations to evaluate short-term intervention effects.

**Results:**

At 3 months following the 2-month integrated intervention program, the intervention group showed significant improvements in self-management (β = 15.51, 95% CI: 5.03–25.99, *P* = 0.004), tuberculosis prevention and control knowledge (β = 0.87,95%*CI*: 0.14–1.60, *P* = 0.019), and chest radiograph improvement rate (χ^2^ = 7.497, *P* = 0.024) compared with the control group. No significant between-group differences were observed in perceived social support (β = −1.09, 95% *CI*: −6.26 to 4.07, *P* = 0.678), anxiety (β = 0.96, 95% *CI*: −0.36 to 2.27, *P* = 0.154), depression (β = 0.13, 95% *CI*: −1.36 to 1.62, *P* = 0.864), or sputum smear and culture conversion rates (χ^2^ = 1.857, *P* = 0.395).

**Conclusions:**

The LEARNS model-based self-management program benefits inpatients with newly diagnosed pulmonary tuberculosis. It effectively improves patients' self-management and tuberculosis prevention and control knowledge, and improves chest radiographic findings. However, this intervention yields no significant effects on anxiety, depression, social support, and sputum bacteriological conversion.

## 1 Introduction

The World Health Organization (WHO) 2025 Global Tuberculosis Report documented 10.7 million new tuberculosis (TB) cases worldwide in 2024, with an incidence rate of 131 per 100,000 population. China reported an estimated 696,000 new TB cases, corresponding to an incidence rate of 49 per 100,000 population, which accounted for 6.5% of the global TB burden and ranked China fourth among the 30 high-TB-burden countries (1). To curb the global TB epidemic, the WHO has promoted short-course chemotherapy strategy centered on directly observed treatment (DOT), leading to a steady annual decline of 2% in global TB incidence (2). However, disadvantages of DOT, including excessive human and financial resource consumption and increased psychological distress caused by privacy exposure, have impeded progress in epidemic control (3). Accordingly, the global TB incidence has failed to meet the WHO's 2025 target of a 50% reduction in disease prevalence (1).

The WHO Global Plan to End Tuberculosis (2023–2030) highlights that people-centered management is pivotal to curbing the global tuberculosis epidemic. As a core patient-centered intervention strategy, self-management serves as a vital complement to Directly Observed Treatment (DOT), and is particularly applicable to patients who are unwilling or unable to access standardized DOT services (4). Nevertheless, newly diagnosed tuberculosis patients, a critical priority population for TB prevention and control, generally present with inadequate disease risk perception, prominent negative emotions, and insufficient family and social support, resulting in poor self-management awareness and capacity (5).

To optimize self-management performance and clinical outcomes in patients with tuberculosis, numerous domestic and international studies have developed and validated various self-management intervention strategies. Based on the Integrated Theory of Health Behavior Change, Bao et al. (6) delivered an online self-management intervention through a WeChat-based platform for hospitalized TB patients, which significantly improved patients' self-management competence, medical service satisfaction, and medication adherence. Guided by the Health Action Process Approach, Chen et al. (5) implemented targeted interventions for older adults TB patients, yielding remarkable improvements in patients' self-management proficienc. Khachadourian et al. (7) further demonstrated that family-participatory health education effectively relieved depressive symptoms and reduced stigma among TB patients.

The aforementioned studies have sufficiently demonstrated that structured self-management interventions can effectively optimize physical and mental wellbeing and improve treatment outcomes among tuberculosis patients. Nevertheless, several notable limitations persist in existing evidence. In terms of study populations, most current interventions primarily target older adults patients and individuals with drug-resistant TB, while tailored strategies for newly diagnosed tuberculosis patients remain understudied. To date, few intervention programs have been specifically designed to address the unique clinical characteristics of newly diagnosed patients, including insufficient early disease cognition, acute emotional distress, and inadequate self-management competencies. Furthermore, conventional intervention approaches are relatively homogeneous and fail to accommodate patients' holistic physical, psychological, and social needs. Traditional health education predominantly adopts unidirectional knowledge delivery, neglecting patient autonomy and active engagement, and lacks sustainable interactive follow-up. As TB prevention and control strategies increasingly shift toward patient-centered care, exploring innovative intervention models that integrate individualization, interactivity, and continuity has become a critical research priority in modern TB nursing.

The LEARNS model is a patient-centered learning framework composed of six iterative stages: Listen, Establish, Adopt, Reinforce, Name, and Strengthen.

The term “LEARNS” is derived from the initial letters of these six core components (8). Unlike conventional monotonous intervention strategies, the LEARNS model effectively addresses the inherent deficiencies of traditional health education, including unidirectional knowledge delivery, rigid procedural protocols, and standardized homogenized intervention modes. Throughout the entire intervention process, the model fully respects patients' subjective will and decision-making autonomy. It comprehensively considers patients' baseline disease cognition, information receptivity, and personalized health demands, and dynamically adjusts educational strategies via a six-stage closed-loop workflow. This iterative design enables patients to gradually establish standardized disease perceptions, correct unhealthy behavioral patterns, and relieve negative psychological emotions, thereby achieving full-cycle, individualized, and precise nursing interventions.

The LEARNS model has been increasingly adopted in chronic disease management and has proven effective in enhancing patients' self-management capacity and therapeutic outcomes. However, its application in newly diagnosed tuberculosis patients remains underreported. Accordingly, this study hypothesizes that, compared with routine nursing care, LEARNS model-based intervention can significantly improve self-management, perceived social support, and TB prevention and control knowledge among newly diagnosed tuberculosis patients, alleviate patients' anxiety and depression symptoms, and optimize clinical treatment outcomes. This study aims to provide a novel and feasible nursing strategy for individualized self-management intervention targeting newly diagnosed tuberculosis patients.

## 2 Methods

### 2.1 Study design and setting

This study adopts a prospective, assessor-blinded, two-arm parallel randomized controlled trial (RCT) design with 1:1 balanced randomization for participant group allocation. Participants with newly diagnosed pulmonary tuberculosis were recruited via convenience sampling from the Tuberculosis Department of a tertiary specialized hospital in Ningxia. This institution is the only tertiary hospital in Ningxia dedicated to pulmonary tuberculosis treatment and runs two separate tuberculosis departments. Each department contains two wards, including 30 rooms and 69 beds in total.

In 2023, the permanent resident population of Ningxia was 7.29 million, and the notified incidence rate of tuberculosis stood at 32.25 per 100,000 population (9).

Eligibility screening was conducted on all newly admitted patients with confirmed tuberculosis between May and July 2024 until the required sample size was achieved.

All enrolled participants were followed up for 5 months after treatment initiation, and the last follow-up for the final participant was completed on December 16, 2024.

### 2.2 Participants

Convenience sampling was used to screen all hospitalized patients with pulmonary tuberculosis admitted to the tuberculosis department of the designated hospital. The inclusion criteria for participants are listed below: (1) Diagnosed with pulmonary tuberculosis (confirmed or clinical diagnosis) in accordance with the national standard WS 288-2017 Diagnosis of Pulmonary Tuberculosis issued by the National Health Commission of the People's Republic of China (10); (2) Newly diagnosed, treatment-naive patients with non-drug-resistant pulmonary tuberculosis; (3) Scheduled to receive a standardized anti-tuberculosis chemotherapy regimen lasting no less than 6 months; (4) Aged between 18 and 75 years old. Exclusion Criteria:

(1) Complicated with other infectious diseases (such as HIV/AIDS and hepatitis B) or extrapulmonary tuberculosis; complicated with severe organic dysfunction involving the heart, lungs, brain, liver or kidneys; accompanied by massive hemoptysis or critical conditions; (2) Suffering from severe visual or auditory impairment, mental disorders or other conditions that hinder normal communication and cooperation.

Dropout Criteria: (1) Occurrence of severe adverse events that make continued intervention inappropriate; (2) Voluntary withdrawal from the study for personal reasons; (3) Loss to follow-up for any cause.

### 2.3 Sample size calculation

For comparison of means between two independent groups, the sample size calculation formula is expressed as: *N*_1_ = *N*_2_ = 2[(μ_α_+μ_β_)/(δ/σ)]^2^ + 0.25$\mu_{a}^{2}$,

where *N*_1_ and *N*_2_ represent the required sample size for each group; δ denotes the difference between the two population means; and σ is the population standard deviation (equal standard deviations are assumed for both groups).

The primary outcome measure in this study is the self-management of chronic disease patients. According to reference (11), post-intervention scores for the observation group and control group were 178.26 ± 15.02 and 81.25 ± 8.75, respectively. Thus, δ = μ1-μ_2_=97.01 and σ = 11.89 (mean of the two sample standard deviations).

We set a two-tailed significance level of α = 0.05 and a Type II error of β = 0.1. After substituting all preset parameters into the sample size formula, the theoretical sample size for each group was far smaller than the generally accepted minimum sample size standard for randomized controlled trials. To ensure the reliability and credibility of research results, we conservatively set the minimum sample size as 30 participants for each group. With a predicted 20% loss to follow-up during the trial, 36 subjects were enrolled per group, leading to an overall total sample size of 72.

### 2.4 Randomization and blinding

After participants provided written informed consent and completed baseline questionnaires, they were randomly allocated to the intervention group or control group at a 1:1 ratio. The random sequence of 72 cases was generated using SPSS 27.0 by an independent researcher who was not involved in participant recruitment, intervention delivery, or outcome assessment. Each allocation outcome was printed on paper slips, placed into sequentially numbered, opaque, sealed envelopes, and securely stored to ensure complete allocation concealment throughout the trial.

Group allocation was performed sequentially by another independent researcher who was not involved in random sequence generation or allocation concealment. Blinding procedures were strictly implemented throughout the trial. Outcome assessors and data analysts remained fully blinded to group allocation throughout the study. Participants and intervention providers were prohibited from revealing group allocation details to blinded study personnel. All personal identifiable information was removed prior to data analysis. The full group allocation sequence was only unblinded by independent researchers upon completion of all statistical analyses. Due to the nature of the behavioral intervention used in this study, blinding of participants and intervention providers was not feasible, and this limitation has been clearly stated in the revised manuscript.

### 2.5 Ethical considerations and study registration

All study procedures were performed in accordance with the ethical principles of the Declaration of Helsinki. This study was approved by the Ethics Committee of Ningxia Medical University (Approval No.: 2024-N180) and was registered in the Chinese Clinical Trial Registry (ChiCTR2400090561) on October 8, 2024. The full trial protocol was finalized prior to participant recruitment in May 2024, and participant enrollment was conducted from May to July 2024. Formal trial registration was completed on October 8, 2024, delayed due to institutional administrative procedures, resulting in retrospective registration. Importantly, no modifications were made to the study design, eligibility criteria, intervention strategies, or primary and secondary outcome measures throughout the entire study period. All prespecified outcomes were reported objectively in full, and no selective outcome reporting was present in this trial.

### 2.6 Intervention protocol

According to the Technical Guidelines for Tuberculosis Prevention and Control in China (2021 Edition), all participants received the standard 6-month anti-tuberculosis regimen of 2HRZE/4HR (H: isoniazid; R: rifampicin; Z: pyrazinamide; E: ethambutol hydrochloride). For patients with persistently positive sputum bacteriology at the end of the 2-month intensive phase, the intensive treatment period was extended by 1 month, while the consolidation phase regimen remained unchanged.

The control group received routine tuberculosis self-management education, covering admission guidance, introduction to the efficacy and adverse reactions of anti-tuberculosis medications, standardized sputum collection, dietary guidance, psychological support, and discharge education.

The intervention group received a standardized self-management intervention for patients with newly diagnosed pulmonary tuberculosis based on the LEARNS (Listen, Establish, Adopt, Reinforce, Name, Strengthen) model. The intervention was delivered by a nurse-led multidisciplinary team comprising eight members: 2 head nurses, 2 responsible nurses, 2 physicians, 1 psychological counselor, and 1 clinical dietitian. The intervention adopted a structured six-step LEARNS framework and focused on three core self-management dimensions: medical management, behavioral management, and emotional regulation. Multiple intervention modalities were adopted, including offline one-on-one guidance, standardized health education manuals, telephone follow-ups, online WeChat consultation, and health education videos. Each intervention session lasted 5–30 min. Intervention duration and frequency were strictly standardized. Patients received 2–5 sessions of offline face-to-face health education during hospitalization. Telephone follow-ups and video interventions were provided for 2 months after discharge, with a total of 4–7 follow-up sessions. Continuous online consultation via the WeChat group was provided throughout the entire treatment course. The core intervention objective was to cultivate patients' self-management awareness and improve their professional knowledge and practical skills for standardized tuberculosis management. The detailed development process and specific intervention contents are shown in Supplementary Table 1, 2

### 2.7 Outcome measurement

#### 2.7.1 Primary outcome measures

##### 2.7.1.1 Self-health management ability evaluation scale for chronic disease patients

The Self-Health Management Ability Evaluation Scale for Chronic Disease Patients was adopted in this study. Developed by Wang (12), this scale is primarily used to evaluate the effectiveness of chronic disease self-management interventions. It consists of 49 items covering five dimensions: cognitive ability, psychological status, behavioral lifestyle, social environment, and treatment adherence. Adopting a five-point Likert scale (never, seldom, sometimes, often, always), each item is scored from 1 to 5. A higher score indicates a higher level of patients' self-management ability. The Cronbach's alpha coefficient of the scale was 0.919.

#### 2.7.2 Secondary outcome measures

**(1) Perceived social support scale (PSSS)**

Developed by Zimet et al. (13) and revised by Jiang (14), the Perceived Social Support Scale (PSSS) consists of 12 items across three dimensions: family support, friend support, and other support. All items are rated on a 7-point Likert scale, with total scores ranging from 12 to 84. Higher scores indicate higher levels of perceived social support. The scale had good internal reliability (Cronbach's α = 0.880).

**(2) Hospital anxiety and depression scales (HADS)**

The Hospital Anxiety and Depression Scale (HADS) was developed by Zigmond and Snaith (15) and translated and revised into the Chinese version by Ye and Xu (16) in 1993. It is widely used to screen anxiety and depression symptoms in patients with somatic diseases. The scale consists of 14 items divided into two subscales: odd-numbered items assess anxiety symptoms, and even-numbered items assess depression symptoms. Each item is scored from 0 to 3 points. Subscale scores are classified as normal (0–7), mild (8–10), moderate (11–14), and severe (15–21). The HADS showed excellent internal consistency (Cronbach's α = 0.919).

**(3) Tuberculosis knowledge questionnaire**

This 11-item questionnaire was developed based on national tuberculosis prevention guidelines, covering core knowledge (e.g., disease characteristics, transmission routes, early symptoms, prevention measures, and policy awareness) and specific practical knowledge (e.g., treatment course, sputum disposal, and treatment precautions). Each correct answer was scored 1 point, while incorrect answers scored 0 points. A total score of ≥7 was defined as adequate disease knowledge. The questionnaire was reviewed by professional tuberculosis experts and exhibited acceptable internal consistency in this study, with a Cronbach's α coefficient of 0.780 (17).

**(4) Treatment efficacy**

This study employed the following indicators to evaluate the therapeutic efficacy of tuberculosis patients after 2 months of intensive treatment, with detailed definitions presented below: Improvement in laboratory parameters: the sputum smear or culture conversion rate at the end of the 2-month treatment period among patients with positive baseline microbiological results. Sputum smears were examined under a fluorescence microscope for the presence of acid-fast bacilli, which is a key diagnostic indicator for pulmonary tuberculosis. Sputum culture was performed using modified Löwenstein-Jensen medium to identify *Mycobacterium tuberculosis*. Chest CT improvement rate: the proportion of patients with partial or complete resolution of tuberculosis lesions on chest computed tomography (CT) at the end of the 2-month intensive anti-tuberculosis treatment phase.

### 2.8 Data collection and quality control

All study data were prospectively collected by dedicated, blinded personnel independent of intervention delivery to ensure data objectivity and completeness.

All research personnel completed standardized pre-study training to standardize operational procedures. A single-case enrollment strategy in individual wards was strictly implemented to eliminate contamination bias. All participants provided full informed consent and signed written consent before enrollment. For participants with lower educational attainment, neutral and non-suggestive questioning was adopted to minimize response bias.

Data collection was performed at three predefined time points. Baseline data (T_0_) were collected through face-to-face paper-based questionnaires during hospitalization. Immediate post-intervention (T_1_) and 3-month follow-up (T_2_) data were collected via telephone interview, WeChat consultation, or online questionnaire following participants' secondary verbal consent. Strict privacy protection measures and standardized follow-up protocols were implemented throughout the study to minimize the loss-to-follow-up rate. All retrieved questionnaires were immediately verified for validity and logical integrity, with invalid data excluded. All raw data were independently double-entered and logically cross-checked by two researchers to avoid input errors. Final valid data were analyzed in strict accordance with standardized statistical protocols.

To ensure intervention consistency within and between groups, all interventionists completed standardized pre-service training, which encompassed the formal LEARNS model-based procedures, core intervention content, communication techniques, follow-up protocols, and standardized answers to frequently encountered questions. Unified intervention manuals and instructional materials were adopted throughout the study to minimize human-induced operational inconsistencies. Specially assigned staff dynamically monitored participant compliance, recorded participation status, and provided proactive follow-up reminders. No modifications were made to the core intervention components throughout the study to ensure standardized, consistent, and reproducible intervention delivery.

### 2.9 Statistical analysis

All data were entered into Excel and analyzed using IBM SPSS Statistics 27.0: IBM Corporation, Armonk, New York, USA. The intention-to-treat (ITT) analysis was applied to evaluate the intervention efficacy. Missing values caused by loss to follow-up or participant withdrawal were handled using the last-observation-carried-forward (LOCF) method. Categorical variables were described as frequencies and percentages, and between-group comparisons were performed using the chi-square test. Normally distributed continuous data were presented as mean ± standard deviation, while non-normally distributed data were reported as median (*Q*_1_, *Q*_3_). The Mann-Whitney *U* test was used for two-group comparisons, and the Wilcoxon test was adopted for pairwise within-group comparisons at each time point. Generalized estimating equations (GEE) were used to evaluate the group, time, and group-by-time interaction effects of the intervention on primary and secondary outcomes. Given three brief follow-up time points and stable within-subject correlations for all outcomes, we adopted the exchangeable (EXCH) working correlation structure for every GEE model. This structure fits longitudinal clinical datasets in which repeated measurements collected from a single participant have comparable correlation strengths, and it produces stable parameter estimates for weakly correlated outcome data. Robust standard errors were applied to covariance estimation to alleviate bias arising from within-subject clustering and secure valid inference despite potential misspecification of the working correlation structure.

Post-fitting residual diagnostics assessing normality and independence confirmed good model fit and that all core GEE assumptions were satisfied. All analyses were two-sided, with a significance level of *a* = 0.05; *P* < 0.05 was considered statistically significant.

## 3 Results

### 3.1 Participants' characteristics

**Figure 1:**
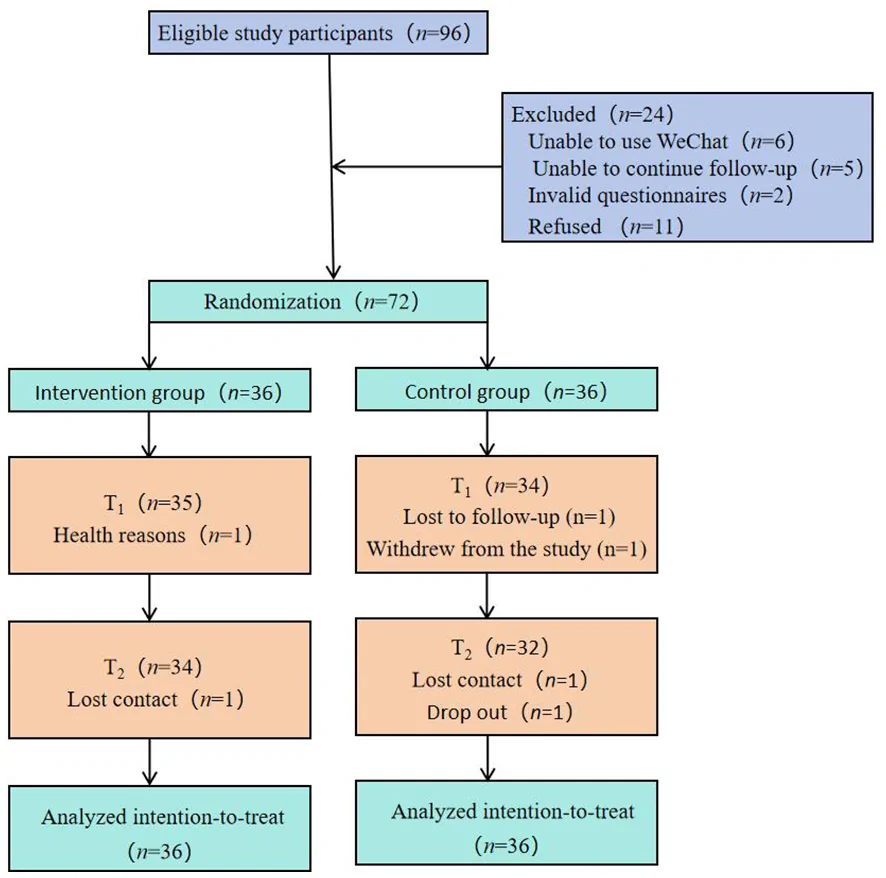
Study participant flowchart.

A total of 96 potential participants were screened for this study from May 16 to July 16, 2024. At baseline (T_0_), a total of 72 eligible participants who met the inclusion criteria signed informed consent forms and were enrolled and randomized after completing baseline assessments. Immediately after the intervention (T_1_), 69 participants finished the follow-up assessment, including 1 dropout in the intervention group and 2 dropouts in the control group. At the 3-month post-intervention follow-up (T_2_), 66 participants completed the assessment, with another 1 dropout in the intervention group and 2 dropouts in the control group. The overall follow-up loss rate throughout the study period was 8.3%. The participant flow across all study stages is presented in Figure 1. The 72 eligible and enrolled participants had a mean age of 42.28 ± 15.75 years; most participants were male (59.7%) and had sputum culture-positive tuberculosis (86.1%). Baseline demographic and clinical characteristics were comparable between the two groups, with no statistically significant differences (*P* > 0.05), as summarized in Table 1.

**Table 1:** Comparison of baseline demographic and clinical characteristics between the two groups (*n*, %).

Variables	Total (*n*=72)	Intervention group *(n*=36)	Control group (*n*=36)	χ^2^	*P*
Gender				0.520	0.471
Male	43 (59.7)	20 (55.6)	23 (63.8)		
Female	29 (40.3)	16 (44.4)	13 (36.1)		
Age (years)				4.250	0.119
18–44	38 (52.8)	19 (52.8)	19 (52.8)		
45–59	18 (25.0)	6 (16.7)	12 (33.3)		
≥60	16 (22.2)	11 (30.6)	5 (13.9)		
Educational attainment				0.661	0.882
Elementary school or below	19 (26.4)	11 (30.6)	8 (22.2)		
Junior high school	23 (31.9)	11 (30.6)	12 (33.3)		
High School/Vocational School	11 (15.3)	5 (13.8)	6 (16.7)		
Associate's degree or higher	19 (26.4)	9 (25.0)	10 (27.8)		
Monthly per capita household income (RMB)				1.048	0.790
<1000	9 (12.5)	5 (13.9)	4 (11.1)		
1000–3000	17 (23.6)	7 (19.4)	10 (27.8)		
3001–5000	25 (34.7)	14 (38.9)	11 (30.6)		
≥5001	21 (29.2)	10 (27.8)	11 (30.6)		
Marital status				0.000	1.000
Unmarried	18 (25.0)	9 (25.0)	9 (25.0)		
Married	52 (72.2)	26 (72.2)	26 (72.2)		
Widowed	2 (2.8)	1 (2.8)	1 (2.8)		
Residency				0.520	0.471
Urban	43 (59.7)	23 (63.9)	20 (55.6)		
Rural	29 (40.3)	13 (36.1)	16 (44.4)		
Other chronic disease (number)				0.077	0.781
0	55 (76.4)	27 (75.0)	28 (77.8)		
≥1	17 (23.6)	9 (25.0)	8 (22.2)		
Sputum smear or culture				1.858	0.173
Negatives	10 (13.9)	3 (8.3)	7 (19.4)		
Positive	62 (86.1)	33 (91.7)	29 (80.6)		

### 3.2 Primary results

GEE analysis revealed a significant main group effect on self-management scores between the two groups (β = 15.51, 95% CI: 5.03–25.99, *P* = 0.004), indicating that the intervention group yielded superior outcomes relative to the control group. A significant main time effect was also observed. Specifically, self-management scores at the 3-month post-intervention follow-up (T_2:_ β = 17.17, 95% CI: 10.64–23.70, *P* < 0.001) were significantly higher than those immediately after the intervention (T_1:_ β = 10.47, 95% CI: 5.23–15.70, *P* < 0.001) suggesting a gradual improvement in patients' self-management levels over time. Additionally, a significant group-by-time interaction effect was identified. The interactive effect was more pronounced at T_2_ (T_2_: β = 32.20, 95% CI: 19.88–44.51, *P* < 0.001) than at T_1_ (T_1_: β = 22.68, 95% CI: 10.99–34.38, *P* < 0.001), demonstrating that the interventional benefits were amplified over time, with the intervention group exhibiting continuously better self-management performance than the control group. Detailed data are presented in Table 2.

**Table 2:** Effects of interventions on subjective outcomes (intention-to-treat analysis).

Variables	Intervention group *(n=*36)	Control group *(n*=36)	*Z*	*P*	Group effect		Time effect		Interaction effects	
					*β (*95% *CI)*	*P* value	*β (*95% *CI)*	*P* value	*B (*95% *CI)*	*P* value
Self-management					15.51 (5.03, 25.99)	0.004				
T_0_	156.00 (145.50, 177.00)	153.00 (141.00, 177.75)	−0.82	0.414						
T_1_	176.00 (165.50, 197.00)	163.00 (141.00, 182.50)	−2.87	0.001			10.47 (5.23, 15.70)	<0.001	22.68 (10.99, 34.38)	<0.001
T_2_	184.50 (173.00, 210.00)	165.44 (152.00, 182.00)	−3.57	<0.001			17.17 (10.64, 23.70)	<0.001	32.20 (19.88, 44.51)	<0.001
PSSS					−1.09 (−6.26, 4.07)	0.678				
T_0_	54.50 (48.00, 66.75)	60.00 (54.00, 65.75)	−1.08	0.279						
T_1_	59.74 (48.25, 70.00)	61.00 (50.25, 68.75)	−0.09	0.933			2.02 (−0.58, 4.61)	0.197	1.47 (−4.35, 7.30)	0.619
T_2_	59.50 (49.00, 66.00)	60.00 (50.75, 66.00)	−0.02	0.982			1.78 (−0.93, 4.49)	0.129	0.29 (−5.44, 6.03)	0.921
Anxiety					0.96 (−0.36, 2.27)	0.154				
T_0_	7.00 (5.00, 9.75)	5.00 (4.00, 8.00)	−1.49	0.135						
T_1_	6.00 (4.00, 8.00)	6.50 (4.00, 8.00)	−0.13	0.896			−0.21 (−1.07, 0.66)	0.638	0.32 (−1.40, 2.03)	0.716
T_2_	5.88 (3.22, 9.00)	5.00 (2.05, 8.00)	−1.16	0.246			−0.91 (−1.84, 0.02)	0.055	0.30 (−1.45, 2.05)	0.735
Depression					0.13 (−1.36, 1.62)	0.864				
T_0_	8.00 (6.25, 10.00)	7.00 (3.25, 9.00)	−0.94	0.350						
T_1_	7.50 (4.00, 9.75)	7.50 (3.50, 11.00)	−0.58	0.560			−0.31 (−1.10, 0.49)	0.450	−0.21 (−1.87, 1.46)	0.809
T_2_	7.00 (2.00, 10.00)	7.00 (5.00, 8.39)	−0.05	0.959			−0.64 (−1.63, 0.35)	0.205	−0.37 (−2.16, 1.41)	0.681
Tuberculosis knowledge					0.87 (0.14, 1.60)	0.019				
T_0_	6.00 (4.00, 8.00)	7.00 (5.00, 8.75)	−0.78	0.433						
T_1_	10.00 (8.25, 11.00)	9.00 (7.25, 10.00)	−2.74	0.006			2.94 (2.42, 3.47)	<0.001	3.42 (2.44, 4.40)	<0.001
T_2_	11.00 (10.01, 11.00)	9.00 (8.00, 10.00)	−5.37	<0.001			3.63 (3.03, 4.22)	<0.001	4.44 (3.55, 5.32)	<0.001

### 3.3 Secondary outcomes

#### 3.3.1 Effect of the intervention on PSSS

GEE analysis was performed to examine the effects of the intervention on perceived social support. The results revealed no significant main group effect (β = −1.09, 95% CI: −6.26 to 4.07, *P* = 0.678) and no significant main time effect (β = 1.78, 95% CI: −0.93 to 4.49, *P* = 0.129). Additionally, the group-by-time interaction effect was also non-significant (β = 0.29, 95% CI: −5.44 to 6.03, *P* = 0.921). Detailed data are presented in Table 2.

#### 3.3.2 Effects of the intervention on anxiety and depression

GEE analysis was conducted to evaluate the intervention effects on anxiety and depression scores. For anxiety outcomes, no significant main group effect (β = 0.96, 95% CI: −0.36 to 2.27, *P* = 0.154), main time effect (β = −0.91, 95% CI: −1.84 to 0.02, *P* = 0.055), or group-by-time interaction effect (β = 0.30, 95% *CI*: −1.45 to 2.05, *P*=0.735) was observed. Similarly, for depression scores, the GEE results also indicated non-significant main group effect (β = 0.13, 95% CI: −1.36 to 1.62, *P* = 0.864), main time effect (β = −0.64, 95% CI: −1.63 to 0.35, *P* = 0.205), and group-by-time interaction effect (β = −0.37, 95% CI: −2.16 to 1.41, *P* = 0.681). These findings suggested that the LEARNS model-based intervention yielded no significant longitudinal effects on improving anxiety and depression symptoms among newly diagnosed tuberculosis patients. Detailed data are presented in Table 2.

#### 3.3.3 Effect of the intervention on tuberculosis knowledge

GEE analysis was performed to evaluate the intervention effects on tuberculosis knowledge scores. Significant main group effects were identified (β = 0.87, 95% CI: 0.14–1.60, *P* = 0.019), demonstrating that the intervention group exhibited superior improvements in tuberculosis knowledge compared with the control group. A significant main time effect was also observed. Specifically, participants obtained higher knowledge scores at the 3-month post-intervention follow-up (T_2:_ β = 3.63, 95% CI: 3.03–4.22, *P* < 0.001) than immediately after the intervention (T_1:_ β = 2.94, 95% CI: 2.42–3.47, *P* < 0.001), indicating a continuous increase in patients' tuberculosis knowledge levels over time. Furthermore, a significant group-by-time interaction effect was detected. The interactive intervention effect was stronger at T_2_ (T_2:_ β = 4.44, 95% CI: 3.55–5.32, *P* < 0.001) than at T_1_ (T_1:_ β = 3.42, 95% CI: 2.44–4.40, *P* < 0.001), suggesting that the LEARNS model-based intervention exerted an amplified longitudinal benefit, with sustained and enhanced improvements in tuberculosis knowledge among newly diagnosed tuberculosis patients in the intervention group. Detailed data are presented in Table 2.

#### 3.3.4 Effect of the intervention on treatment outcome measures

**(1) Comparison of sputum conversion between the two groups**

Chi-square test was adopted to compare T_1_ sputum smear or culture conversion rates among patients with baseline positive sputum bacteriology. Valid sputum samples were unavailable at the T_2_ follow-up for most participants; thus, only T_1_ bacteriological outcomes were analyzed. The results demonstrated no significant between-group difference in sputum conversion rates (*P* > 0.05). Detailed data are presented in Table 3.

**Table 3:** Comparison of sputum smear or culture conversion rates between the two groups (*n*, %).

Sputum conversion	Intervention group *(n* = 32)	Control group *(n* = 29)	χ^2^	*P*
No sputum sample was collected	17 (53.1)	17 (58.6)	1.857	0.395
Positive for sputum culture	0 (0)	1 (3.5)		
Sputum culture turned negative	15 (46.9)	11 (37.9)		

**(2) Comparison of chest CT radiological improvement between the two groups**

Chi-square analysis was performed to compare chest CT radiological outcomes at the T_1_ time point between the two groups. Since most participants did not undergo follow-up chest CT examination at T_2_, only T_1_ imaging data were analyzed in the present study. At the T_1_ post-intervention assessment, pulmonary lesion shrinkage was observed in 82.9% (29/35) of patients in the intervention group, compared with 55.9% (19/34) in the control group. A significant between-group difference in radiological improvement was identified (*P* < 0.05), suggesting that the intervention yielded superior effects on the remission of pulmonary lesions. Detailed data are presented in Table 4.

**Table 4:** Comparison of chest CT radiological improvement between the two groups (*n*, %).

Chest CT scan	Intervention group *(n* = 35)	Control group (*n* = 34)	χ^2^	*P*
Lesion shrinkage	29 (82.9)	19 (55.9)	7.497	0.024
No significant change	6 (17.1)	13 (38.2)		
Not examined	0 (0)	2 (5.9)		

## 4 Discussion

This randomized controlled trial developed and implemented a 2-month LEARNS model-based self-management education program and systematically evaluated its short-term therapeutic effects among newly diagnosed tuberculosis patients. Generalized estimating equation analyses revealed that this tailored educational intervention significantly and continuously improved patients' disease self-management and tuberculosis-related knowledge, leading to superior short-term radiological lesion resolution. Nevertheless, no significant between-group differences were identified in anxiety, depressive, or perceived social support after the intervention. Collectively, these findings indicate that LEARNS model-based self-management education exerts targeted and robust benefits on patient behavioral self-regulation, disease cognition, and short-term clinical therapeutic effects, whereas a 2-month educational regimen is insufficient to improve patients' psychological status and social support perception.

The enhanced self-management and disease cognition observed in the intervention group validate the clinical applicability of LEARNS model-based self-management education among patients with chronic infectious diseases, which aligns with the findings of prior LEARNS behavioral intervention studies (18). The patient-centered core concept of the LEARNS model is highly compatible with the evidence-based behavioral counseling framework summarized by Whitlock et al. (19).

This study synthesized classic health behavior change theories and standardized operational procedures for clinical behavioral interventions, clarifying that sustained behavioral improvement depends on individualized assessment and active patient engagement rather than passive knowledge indoctrination. Individualized behavioral education centered on active patient engagement serves as a vital non-pharmacological strategy to optimize early therapeutic responses among patients with pulmonary tuberculosis (20). The full-course intervention framework of the LEARNS model offers a standardized and implementable clinical paradigm. It enables newly diagnosed tuberculosis patients to conduct proactive disease management and achieve better short-term therapeutic outcomes. Accumulated evidence has verified that structured and systematic self-management education can standardize patients' treatment behaviors. It reduces the adverse impacts of unhealthy lifestyles on treatment and accelerates early pulmonary lesion absorption (6).

Solid mastery of tuberculosis-related knowledge constitutes a core cognitive prerequisite for standardized self-management and optimized short-term therapeutic outcomes among pulmonary tuberculosis patients. Newly diagnosed patients often present fragmented and unstable disease cognition. Such cognition gradually declines during treatment, acting as a key implicit barrier to early treatment compliance.

The present study demonstrated that the LEARNS model-based intervention effectively improved tuberculosis knowledge proficiency among newly diagnosed patients. Patients' cognitive level increased continuously during the intervention and achieved better efficacy than routine nursing, which is consistent with previous studies (21). Traditional health education adopts one-time centralized lectures. This mode easily leads to knowledge decay and residual cognitive bias. In contrast, the whole-course and progressive cognitive intervention in this study continuously corrects patients' cognitive misunderstandings and remedies knowledge deficiencies. It helps patients establish stable and systematic disease cognition, which accounts for the sustained cognitive improvement in the intervention group. Improved disease knowledge further exerts positive effects on treatment compliance, treatment completion rate, and cure rate among tuberculosis patients (22).

The LEARNS model-based self-management intervention effectively promotes pulmonary lesion absorption, optimizes early radiological outcomes, and improves short-term therapeutic effects in newly diagnosed pulmonary tuberculosis patients, consistent with previous evidence (23). The favorable intervention effects can be illustrated in three aspects. First, the LEARNS intervention covers the full treatment period from hospitalization to the end of intensive therapy. Adopting a multi-modal strategy including face-to-face guidance, printed educational booklets, telephone and WeChat follow-up, and short-video-based health education, this integrated digital intervention effectively reduces treatment interruption. Digital health interventions have been well documented to improve treatment compliance and clinical outcomes in tuberculosis patients (24). Second, standardized lifestyle guidance provided through regular follow-up accelerates disease rehabilitation and alleviates common adverse reactions induced by anti-tuberculosis drugs, including gastrointestinal discomfort, liver injury and hyperuricemia, thereby reducing unauthorized treatment discontinuation caused by physical symptoms. Lifestyle modifications including improved body weight, better sleep quality, and smoking and alcohol cessation have been proven to optimize tuberculosis treatment outcomes (25–27). Third, this study constructed a multi-dimensional “family-community-medical staff” collaborative intervention system. Family members were involved in whole-process health education and supervision, which effectively strengthened patients' adherence to regular medication and outpatient follow-up. Meanwhile, encouraging patients to participate in community standardized tuberculosis management further enriched their social support resources. Existing evidence confirms that diverse supportive models can steadily improve treatment adherence and facilitate successful tuberculosis treatment across different economic settings (28). Although radiological improvement is a core, intuitive indicator for evaluating tuberculosis treatment efficacy, this study only collected two-month staged data during intensive therapy without full-course follow-up, which limits the assessment of patients' definitive long-term outcomes.

No significant intergroup differences in anxiety, depression and social support were found between the two groups. This finding indicates that the LEARNS intervention yields effects comparable to routine nursing in improving patients' psychological symptoms and social support, with no additional therapeutic advantages. Two potential reasons explain these non-significant outcomes. First, all patients were still receiving anti-tuberculosis treatment at follow-up assessment. Tuberculosis-related stigma is prevalent throughout the treatment period, which may impair patients' mental wellbeing and reduce the utilization of social support, thereby limiting improvements in psychological outcomes. Second, psychological symptoms and perceived social support are relatively stable personal traits, which are difficult to substantially improve through a 2-month short-term intervention.

### 4.1 Innovations and limitations

Integrating patient-centered self-management concepts with learning intervention strategies, this study developed a LEARNS model-based self-management education program tailored to newly diagnosed pulmonary tuberculosis patients. Unlike the traditional simplistic and passive health education models widely used in domestic studies, this program integrates individualized guidance with continuous full-course follow-up interventions. It aligns with the patient-centered care principles advocated by current global tuberculosis guidelines. This study further complements the international empirical research system of the LEARNS model for infectious chronic diseases and provides a low-cost, feasible, and scalable non-pharmacological intervention strategy for the early clinical management of pulmonary tuberculosis.

This study presents several limitations to be resolved in subsequent research. First, due to limited study duration, the intervention and follow-up period for patients with newly diagnosed pulmonary tuberculosis was restricted to 2 months. While we could document short-term clinical benefits of the LEARNS model-based intervention, sustained long-term reductions in psychological distress and persistent gains in perceived social support could not be captured, so the long-term efficacy of this intervention remains unestablished. Second, this single-blind trial only masked data collectors; intervention deliverers and participants were unblinded. This design introduces inherent risks of performance bias and response bias, which may inflate the true intervention effect size. While intention-to-treat analysis and objective imaging endpoints were utilized to partially mitigate such bias, residual confounding factors could not be fully ruled out. Third, last observation carried forward (LOCF) was uniformly applied to address longitudinal missing data in the current study. As a traditional missing-data handling strategy, LOCF has inherent statistical drawbacks: it fixes the final observed values of participants lost to follow-up and ignores natural temporal variations in outcome measures, which may yield biased estimates and either underestimate or overestimate intervention effects. Constrained by the characteristics of our dataset, we did not conduct supplementary sensitivity analyses using the more robust multiple imputation method. Fourth, the LEARNS model-based intervention effectively reduced treatment interruptions, promoted medication adherence, and yielded favorable short-term chest radiographic outcomes. However, critical hard endpoints—quantitative adherence measurements and long-term cure rates—were not formally quantified in our analyses. All conclusions about clinical benefits are supported only by interim radiographic improvements. In conclusion, the LEARNS model-based self-management intervention demonstrates robust short-term efficacy, while its long-term therapeutic effects and multi-dimensional quantitative benefits require further validation. Future large-scale, multi-center controlled trials with extended follow-up are needed. Such trials ought to integrate standardized objective measures of medication adherence and long-term cure outcomes, together with a refined full-cycle dynamic intervention and digital monitoring system. Additionally, implementation studies can be carried out to develop context-adapted delivery approaches for primary care facilities, thereby improving the clinical feasibility, stability, and generalizability of the LEARNS model.

## 5 Conclusion

A LEARNS model-based self-management program can effectively improve self-management competence and tuberculosis knowledge among patients with newly diagnosed pulmonary tuberculosis, thereby optimizing their clinical treatment outcomes. Nevertheless, this intervention exerts no significant effects on patients' anxiety, depression, and perceived social support. The findings of this study provide valuable practical evidence and implications for clinical tuberculosis nursing, public health prevention and control, and relevant academic research.

At the clinical application level, the nurse-led LEARNS model-based self-management intervention targets the core clinical problem of inadequate self-management among patients with newly diagnosed pulmonary tuberculosis, establishing a low-cost, standardized, and replicable nursing intervention protocol.

At the public health level, by enhancing patients' self-management, this intervention has the potential to optimize the therapeutic outcomes of pulmonary tuberculosis, facilitate the attainment of national tuberculosis prevention and control targets, and generate substantial public health value by alleviating the overall disease burden of tuberculosis. From an academic perspective, this study expands the application scope of the LEARNS model to tuberculosis management. It comprehensively reports all prespecified study outcomes, including non-statistically significant negative results, thereby offering transparent and credible references for subsequent relevant intervention research.

## Data Availability

The raw data supporting the conclusions of this article will be made available by the authors, without undue reservation.
