# Supplementary material for: Effects of the LEARNS model-based self-management education on short-term treatment outcomes among newly diagnosed tuberculosis patients: a randomized controlled trial
Source: Front Public Health. 2026 Aug 11;14:1861683. doi: 10.3389/fpubh.2026.1861683 (PMC13503355; doi:10.3389/fpubh.2026.1861683)
Supplement: Supplementary file 1 [file Table_1.doc]

**Supplementary Material**

**Protocol Development**

The development process of the intervention protocol for the intervention group was as follows. First, a mixed-methods study with an explanatory sequential design was conducted to investigate and analyze the status of self-management and its influencing factors among newly diagnosed tuberculosis patients. Quantitative findings showed that educational level, social support, anxiety, and knowledge of tuberculosis prevention and control were influencing factors of self-management. Qualitative analysis was structured around the three core dimensions of self-management: medical management, behavioral management, and emotional management. For the medical management dimension, key findings included insufficient medical management knowledge, heavy medication management burden, and associated influencing factors (inadequate family support, lack of continuous professional support, and high financial burden). For the behavioral management dimension, main issues were low self-management awareness, inadequate nutritional intake, fatigue and insomnia, and insufficient disinfection and isolation practices. For the emotional management dimension, identified problems included anxiety and depression, disease-related stigma, and diminished sense of self-worth. Subsequently, based on the above quantitative and qualitative results, the LEARNS model, and a literature review, a draft self-management intervention scheme for newly diagnosed tuberculosis patients was developed. The final version of the LEARNS model-based self-management program for this population was finalized after optimization via the Delphi expert consultation method.

**Supplementary Table 1 Implementation Plan for the Self-Management Program for Newly Diagnosed Tuberculosis Patients Based on the LEARNS Model**

| Intervention Process | Intervention  Time | Intervention Topic/Module | Intervention Method | Intervention Format | Location | Implementer | Duration of Intervention |
| --- | --- | --- | --- | --- | --- | --- | --- |
| Listening | Admission  Same day | Assessment and follow-up | 1.Understand the patient’s basic information and medical condition. | Review medical records | Nurses' Station | Nurse | 15-30 min |
|  |  |  | 2.Understand the patient’s medical history, beliefs, learning needs, and preferences. The interview outline includes: ①How are you feeling right now? (Encourage the patient to express their inner pain, worries, fears, anxiety, confusion, and other feelings; at this point, nurses should be careful to avoid subjectively guiding the conversation, which could lead to inaccurate information); ②Regarding tuberculosis, what do you know about the disease and self-management? ③What other aspects of the condition would you like to learn about? ④What is your preferred method for learning new information? | Semi-structured Interview | At the bedside or in an empty ward |  |  |
|  |  |  | 3.Use the Hospital Anxiety and Depression Scale to assess the patient’s psychological state; use the Social Support Scale to evaluate the patient’s social support status (family support, friend support, other support). | Questionnaire Survey Method |  |  |  |
|  |  | Emotional Management: Psychological Care | 4.Based on a comprehensive assessment of the patient’s emotional issues derived from conversations and questionnaire results, the assigned nurse provides psychological care for patients with mild anxiety or depression. For patients with moderate to severe anxiety or depression, a psychological counselor should be consulted for intervention. | Face-to-face |  | Nurse, Psychologist |  |
|  |  | Medical Management: Family Support | 5.Based on the results of the social support assessment and communication with patients, prioritize patients without family support or those with low to moderate social support scores. For patients with family members, include family members in the education plan. |  |  | Nurse | 5 min |
| Establish | Days 1–2 after admission | Develop a Learning Plan | Based on a thorough understanding of the patient and their learning needs, establish a therapeutic partnership with them. Collaborate with the patient to develop a learning plan, determining the sequence of learning topics as well as the time, location, and method of instruction. | Face-to-face | At the Bedside | Nurse | 10-20 min |

**Supplementary Table 1 (Continued) Self-Management Plan for Newly Diagnosed Tuberculosis Patients Based on the LEARNS Model**

| Intervention Process | Intervention  Time | Intervention Topic/Module | Intervention Method | Intervention Format | Location | Implementer | Duration of Intervention |
| --- | --- | --- | --- | --- | --- | --- | --- |
| Application and Improvement | From the 2nd day of admission to the day before discharge | 1.Medical Management: Health education.  2.Behavioral Management: Raising awareness, healthy eating, sleep and rest, disinfection and isolation. | Based on the listening results, conduct individualized health education sessions tailored to the patient’s preferred method, time, and location. During each session, use plain language appropriate to the patient’s educational background and comprehension level, incorporating examples and images as much as possible.Speak at a moderate pace, observe the patient’s learning response, and adjust the presentation accordingly. Actively guide patients to identify, ask about, and resolve issues, and provide opportunities for hands-on practice, such as the seven-step handwashing method, cough etiquette, and proper mask-wearing. | Face-to-face  Health education, manuals, videos | Bedside | Doctors, nurses, dietitians | 2 sessions, 15-20 minutes each |
| Feedback-based instruction | Day 3 of admission to the day of discharge |  | To ensure patients understand the information they have learned, various methods such as feedback-based instruction, question-answer-question sessions, and student presentations can be used to evaluate learning outcomes. Through feedback-based instruction, the knowledge and skills patients have mastered are clarified and reinforced, while concepts they do not understand or skills they have not mastered are explained again. | Face-to-face, educational videos | Bedside | Nurse | 5-10 minutes per session |
| Reinforcement | Post-discharge | Medical management, Policy support,  Ongoing support. | 1.Post health education content in the WeChat group once a week to reinforce treatment precautions and encourage patients to study the material. | Health education videos |  | Nurse | 5 min |
|  |  |  | 2.Provide ongoing consultation in the WeChat group until the patient completes their treatment course. | WeChat |  | Multidisciplinary Team | On-demand |
|  |  |  | 3.Conduct a telephone follow-up every two weeks until the end of the intensive phase (2 months). | Phone |  | Nurse | 10-40 min |
