# Supplementary material for: Effects of the LEARNS model-based self-management education on short-term treatment outcomes among newly diagnosed tuberculosis patients: a randomized controlled trial
Source: Front Public Health. 2026 Aug 11;14:1861683. doi: 10.3389/fpubh.2026.1861683 (PMC13503355; doi:10.3389/fpubh.2026.1861683)
Supplement: Supplementary file 2 [file Table_2.doc]

**Supplementary Table 2 Intervention Themes and Modules of the Self-Management Program for Newly Diagnosed Tuberculosis Patients Based on the Program Development Framework**

| Intervention Theme | Intervention Module | Intervention Strategies |
| --- | --- | --- |
| Medical Management | Knowledge Education | 1.Basic knowledge of tuberculosis: definition, causes, clinical manifestations, modes of transmission, and preventive measures. |
|  |  | 2.Methods for coughing up sputum, precautions, and skills for collecting sputum specimens. |
|  |  | 3.Symptom management techniques and methods. |
|  |  | 6.Identification and management of hemoptysis. |
|  |  | 5.Principles and duration of anti-tuberculosis treatment. |
|  |  | 6.Types of anti-tuberculosis drugs, adverse reactions, their identification and management, and medication precautions. |
|  | Family Support | 1.Assessing family and social support. |
|  |  | 2.Emphasize the importance of family support for patient self-management; invite family members to participate in patient self-management education and teach them how to provide practical, therapeutic, and psychological support. |
|  |  | 3.For patients without family support, nurses should provide close attention and offer multidimensional support and assistance during their hospital stay. |
|  |  | 4.Inquire with family members about the patient’s medication adherence and any drug side effects. |
|  | Policy Support | Provide patients with information on tuberculosis-related health insurance and subsidy policies, as well as how to access them. |
|  | Follow-up Support | 1.After discharge, encourage patients to enroll in the tuberculosis management program at their local community health center. |
|  |  | 2.Remind patients of their follow-up appointment dates, the specific tests involved, and the importance and significance of these appointments. |
|  |  | 3.Assess and identify issues and difficulties patients face in self-management, and provide personalized guidance and strategies to address these challenges. |
|  |  | 4.Instruct patients on self-management strategies and precautions in their daily work, life, and social interactions to promote self-management. |
|  |  | 5.Timing, methods, and benefits of exercise and rehabilitation. |
|  |  | 6.Identify and manage adverse drug reactions; monitor symptoms such as weight changes and hemoptysis. |
| Behavioral Management | Building Awareness | 1.The concept, purpose, and significance of self-management. |
|  |  | 2.Explain the effectiveness of standardized tuberculosis treatment and the adverse effects of non-standardized treatment to help patients recognize the necessity of actively engaging in self-management. |
|  |  | 3.Provide examples of how self-management promotes recovery to enhance patients’ motivation for self-management. |
|  | Healthy Diet | 1.Balanced Diet: Importance, basic principles, food selection, and dietary restrictions. |
|  |  | 2.Measure weight weekly to monitor the patient’s nutritional status. |
|  | Sleep and Rest | 1.Explain to patients the importance of a healthy lifestyle in promoting successful tuberculosis treatment, including: avoiding late nights to ensure adequate sleep, avoiding overexertion, and quitting smoking and alcohol. |
|  |  | 2.Establish and self-monitor daily health behaviors. |
|  | Infection Control | 1.Educate patients on cough etiquette, sputum management, and hand hygiene. |
|  |  | 2.Instruct patients on the selection and proper wearing of masks. |
|  |  | 3.Home ventilation, disinfection, protective measures, and developing good hygiene habits. |
| Emotional Management | Psychological Care | 1.When receiving patients, nurses should speak to them in a friendly, gentle, and warm manner to alleviate their anxiety and fear. |
|  |  | 2.Assess patients’ negative emotions through conversation and the Hospital Anxiety and Depression Scale (HADS). |
|  |  | 3.Recognize the relationship between positive emotions and disease outcomes; listen to, understand, and support patients to enhance their motivation for self-management. |
|  |  | 4. Communicate frequently with patients to help them maintain a positive mindset and actively cooperate with treatment. |
|  |  | 5.Address stigma and common misconceptions associated with tuberculosis. |
